# Changes in drinking days among United States adults during the COVID‐19 pandemic

**DOI:** 10.1111/add.15622

**Published:** 2021-07-12

**Authors:** Courtney D. Nordeck, Kira E. Riehm, Emily J. Smail, Calliope Holingue, Jeremy C. Kane, Renee M. Johnson, Cindy B. Veldhuis, Luther G. Kalb, Elizabeth A. Stuart, Frauke Kreuter, Johannes Thrul

**Affiliations:** ^1^ Department of Mental Health, Bloomberg School of Public Health Johns Hopkins University Baltimore MD USA; ^2^ Department of Neuropsychology, Kennedy Krieger Institute Johns Hopkins University Baltimore MD USA; ^3^ Department of Epidemiology, Mailman School of Public Health Columbia University New York NY USA; ^4^ School of Nursing Columbia University New York NY USA; ^5^ Joint Program in Survey Methodology University of Maryland MD USA; ^6^ Department of Statistics Ludwig Maximilian University of Munich Munich Germany; ^7^ Statistical Methods Group Institute for Employment Research Nuremberg Germany; ^8^ Centre for Alcohol Policy Research La Trobe University Bundoora VIC Australia

**Keywords:** Alcohol use, COVID‐19, drinking behavior, drinking days, sociodemographic disparities, substance use epidemiology

## Abstract

**Aims:**

To examine changes in drinking behavior among United States (US) adults between March 10 and July 21, 2020, a critical period during the COVID‐19 pandemic.

**Design:**

Longitudinal, internet‐based panel survey.

**Setting:**

The Understanding America Study (UAS), a nationally representative panel of US adults age 18 or older.

**Participants:**

A total of 4298 US adults who reported alcohol use.

**Measurements:**

Changes in number of reported drinking days from March 11, 2020 through July 21, 2020 in the overall sample and stratified by sex, age, race/ethnicity, household structure, poverty status, and census region.

**Findings:**

Compared with March 11, the number of drinking days per week was significantly higher on April 1 by an average of 0.36 days (95% CI = 0.30, 0.43), on May 1 by an average of 0.55 days (95% CI = 0.47, 0.63), on June 1 by an average of 0.41 days (95% CI = 0.33, 0.49), and on July 1 by an average of 0.39 days (95% CI = 0.31, 0.48). Males, White participants, and older adults reported sustained increases in drinking days, whereas female participants and individuals living under the federal poverty line had attenuated drinking days in the latter part of the study period.

**Conclusions:**

Between March and mid‐July 2020, adults in the United States reported increases in the number of drinking days, with sustained increases observed among males, White participants, and older adults.

## Introduction

The coronavirus (COVID‐19) pandemic is an international emergency that has dramatically changed daily life. This global pandemic is expected to have lasting effects on individual well‐being including increased prevalence of psychological distress [1, 2, 3]. The pandemic has resulted in numerous stressors, including social isolation [4] and historically high unemployment rates [5], which are likely to have ongoing implications for public health in the United States (US).

One possible implication of the COVID‐19 pandemic is changes in alcohol use in the general population. Alcohol use, including high‐risk drinking, has increased in the United States over the past decade, particularly among females, older adults, racial/ethnic minorities, sexual minorities, and individuals with lower income [6], highlighting important sociodemographic differences. Because alcohol use is associated with stressful life events [7] and is associated with depression, anxiety, and substance use disorders [8, 9, 10], there are particular concerns regarding alcohol consumption during the COVID‐19 pandemic. Social distancing protocols and stay‐at‐home orders may increase alcohol craving, consumption, and risk of relapse [11, 12, 13]. Indeed, emerging cross‐sectional data have indicated increases in alcohol use in the United States, similar to evidence of increased consumption in Europe [14, 15], China [16], and Australia [17]. Studies of US adults have found significant increases in the frequency of alcohol consumption [18], including binge drinking [19]. Moreover, although some have found evidence of an association between COVID‐19‐related stress and increased drinking behaviors [20], others have found increases in drinking behavior among individuals living in states with relatively lower COVID‐19 disease burden [21], suggesting alcohol use may be sensitive to contextual and psychosocial factors. Finally, there have been increases in alcohol retail sales because many states closed bars/restaurants and relaxed alcohol sale restrictions by allowing curbside distribution or delivery. Although there are expected increases in alcohol sales related to seasonal trends, reported increases in retail sales during the first half of 2020 substantially exceeded similar periods in previous years [22], with online sales increasing 234% compared to 2019 [23].

Collectively, these findings suggest that there may be increases in alcohol consumption during the COVID‐19 pandemic, but this evidence has largely been limited to cross‐sectional and ecological analyses and it remains unclear whether there are sustained increases in use. Therefore, it is important to examine changes in drinking behavior over time and identify sociodemographic subgroups that may be especially at risk for adverse outcomes. To address this gap, the objectives of the current study were (i) to examine changes in number of drinking days from March 10, 2020 through July 21, 2020 among a nationally representative cohort of US adults who reported any alcohol use during the survey period, and (ii) to determine whether trajectories of drinking behavior differed among key sociodemographic subgroups.

## Methods

### Participants

Participants were drawn from the Understanding America Study (UAS), a probability‐based, nationally representative, internet panel of adults (18‐years and older). This study used data from nine waves of the UAS; the baseline wave was conducted from March 10, 2020 to March 31, 2020, and follow‐up waves were conducted thereafter at 2‐week intervals between April 1, 2020 and July 21, 2020. UAS participants were selected using address based sampling (ABS), in which postal records are used to select a random sample from a listing of residential addresses. The recruitment involves several steps, including prepaid and conditional incentives and several reminders. Potential participants without prior internet access are provided with tablets and broadband internet connections. Once respondents have joined the panel, they are surveyed via computer, mobile device, or tablet. Additional details regarding the UAS methodology can be found at the UAS website (https://UASdata.usc.edu).

The baseline wave of data collection consisted of a tracking survey fielded on March 10, 2020; respondents had until March 31, 2020 to complete the survey. Starting on April 1, 2020, respondents were invited to consent to participate in bi‐weekly surveys according to a staggered schedule, whereby one‐fourteenth of the sample was invited every day. Because every respondent has 14 days to complete the survey, the waves overlap in calendar time. Only those respondents who consented were then invited to complete a survey on their assigned day. Because not all eligible participants had yet consented at the start of the second wave, the response rate as a percentage of the complete UAS sample was lower in earlier follow‐ups.

Overall, there were 8547 eligible panel members. We restricted our analytic sample to those participants who reported at least 1 day of alcohol use across the survey period. Additionally, given the low proportion of missing data at each survey (<7%), we included only complete cases at each time point in our analyses, meaning that data were not missing for any of the identified variables. Altogether, 4298 unique participants were included; 62.2% completed nine surveys, 15.1% completed eight surveys, 7.2% completed seven surveys, and the remaining 15.5% completed between one and six surveys (see Supporting information Table S1 for number of observations per day). Supporting information Figure S1 details participant inclusion, response rates, and the proportion of complete observations at each survey, and Supporting information Table S2 presents comparisons between participants who completed all surveys to those who completed 8 or fewer surveys. Comparisons between those who reported any alcohol use and those who reported no use across the study period are displayed in Supporting information Table S3.

### Measures

#### Number of drinking days

The outcome of interest was the number of reported drinking days in the past week at each wave. Participants were provided with a pre‐specified list of activities and asked, “Out of the past 7 days, what is your best estimate of the number of days that you did each of the following activities?” From the list of activities, we used responses for the activity, “Consumed alcohol.” Responses ranged from 0 (alcohol consumed on none of the past 7 days) to 7 (alcohol consumed on all of the past 7 days). Number of reported drinking days was selected as the outcome of interest because this measure was consistently assessed at each wave during the study period.

#### Survey date

We used survey date as the time scale to assess changes over time. Survey date was entered into each model as a continuous variable representing the number of days since March 10, 2020, ending on July 21, 2020 (range, 0–133 days). Given evidence of non‐linear changes in the number of drinking days over time, we modeled survey date with restricted cubic splines, which generate smoothed curves for the relationship between continuous exposures and outcomes. Cubic splines capture features that may be missed by traditional techniques such as linear models or categorization into bins [24]. We generated splines with five knots using the percentiles recommended by Harrell (5, 27.5, 50, 72.5, and 95) to allow for greater variability in modelling and for more flexible interpretation of these non‐linear trends [25]. The knots corresponded to the following dates: March 12 (day 2), April 22 (day 43), May 20 (day 71), June 17 (day 99), and July 15 (day 127).

Sociodemographic characteristics were measured at baseline as time‐fixed variables. These included age (18–29, 30–49, 50–64, or 65+), sex (female or male), race/ethnicity (non‐Hispanic White, non‐Hispanic Black, Hispanic/Latino of any race, or other [American Indian or Alaskan Native, Asian, Pacific Islander, or Multi‐racial]), and state of residence classified according to census regions (Midwest, South, West, and Northeast). We also included an indicator for whether an individual was living in a household above or below the Federal poverty line (FPL). Data for annual household income were recorded in categories; we calculated the median for each category and divided this by the number of individuals in the household to estimate the income per household member. This was used to classify individuals as living in a household above or below the poverty line according to the 2020 Federal Poverty Guidelines. We also included a variable reflecting household structure. Respondents reported their relationships to other household members, as well as the ages of those members; we used this to classify individuals into five categories of household structure (living alone, living with a partner only, living with a partner and children, living with children only, and other [such as living with parents or other relatives, and living with non‐relatives]).

### Statistical analysis

#### Association of date and sociodemographic characteristics with drinking days

We used mixed‐effects linear regression models with a random effect for participant to accommodate repeated measures. Analyses were conducted in three stages. First, we estimated a series of models to examine the association of each sociodemographic characteristic with the average number of drinking days across the entire survey period. Second, we estimated a single model with the splines for days since March 10, 2020, as covariates to examine the trajectory of drinking days over time among all US adults. Third, we estimated a series of models with interactions between the splines for days since March 10, 2020, and each of the identified sociodemographic characteristics to determine whether trajectories of drinking days over time differed between sociodemographic subgroups. Wald tests were used to determine if interactions were statistically significant. The *margins* and the *xbrcspline* commands in Stata were used to generate linear predictions of drinking days and to estimate differences in the number of drinking days on given survey dates compared to March 11, 2020, respectively, in the overall sample and stratified by each sociodemographic subgroup [26]. March 11, 2020 was used as the reference date instead of March 10, 2020 because of a higher number of observations (1430 vs 240, respectively).

To test the sensitivity of our findings to the exclusion of non‐drinkers, we re‐estimated our models in the complete sample of drinkers and non‐drinkers across the study period.

All analyses incorporated survey weights that account for probabilities of sample selection and survey non‐response and are aligned with Current Population Survey benchmarks. Missing observations because of survey non‐response were handled with full information maximum likelihood estimation. Statistical significance was assessed at the *P* < 0.05 level. Analyses were conducted using Stata version 16 (StataCorp) and R (R studio version 1.2.5042; R version 4.0.0). This analysis was not pre‐registered and results presented in this study should be considered exploratory.

## Results

Across the study period, the overall average number of drinking days among participants who reported alcohol use was 2.23 days (95% CI = 2.19, 2.26) in the past 7 days.

### Associations of sociodemographic characteristics and number of drinking days

Sample characteristics and differences in the number of drinking days across the study period are reported in Table 1. The number of drinking days was lower among females (β = −0.79; 95% CI = −0.92, −0.67) compared to males; Black (β = −0.78; 95% CI = −0.99, −0.57), Hispanic/Latino (β = −1.11; 95% CI = −1.25, −0.97), and participants in the other race/ethnicity group (β = −0.84; 95% CI = −1.03, −0.64) compared to White respondents; adults living alone (β = −0.42; 95% CI = −0.63, −0.22), with a partner and children (β = −0.65; 95% CI = −0.82, −0.48), with children only (β = −0.86; 95% CI = −1.16, −0.57), and in other household structures (β = −0.89; 95% CI = −1.06, −0.72), compared to adults living with a partner only; and in adults living at or below the FPL (β = −0.74; 95% CI = −0.92, −0.55), compared to above the FPL. The number of drinking days was higher in older age groups (30–49: β = 0.48; 95% CI = 0.32, 0.63; 50–64: β = 0.79; 95% CI = 0.62, 0.97; 65+: β = 1.41; 95% CI = 1.20, 1.63) compared to those ages 18 to 29. No significant differences in the number of drinking days were observed between US census regions.

**Table 1:** Descriptive statistics of sample characteristics for US adults at the first survey wave and associations with number of reported drinking days across the survey period (*n* = 4298).

*Variable*	*n (%)*	β *(95% CI)*
Sex		
Male	1889 (50.6)	ref.
Female	2409 (49.4)	**−0.79 (−0.92, −0.67)**
Age (in years)		
18–29	522 (13.1)	ref.
30–49	1652 (42.1)	**0.48 (0.32,0.63)**
50–64	1261 (26.5)	**0.79 (0.62,0.97)**
65+	863 (18.3)	**1.41 (1.20,1.63)**
Race		
White	2910 (64.5)	ref.
Black	307 (11.3)	**−0.78 (−0.99, −0.57)**
Hispanic/Latino	680 (16.2)	**−1.11 (−1.25, −0.97)**
Other	401 (8.1)	**−0.84 (−1.03, −‐0.64)**
Household structure		
With partner only	1324 (29.8)	ref.
Alone	715 (15.6)	**−0.42 (−0.63, −0.22)**
With partner and kids	1077 (26.3)	**−0.65 (−0.82, −0.48)**
With kids only	182 (4.2)	**−0.86 (−1.16, −0.57)**
Other	1000 (23.6)	**−0.89 (−1.06, −0.72)**
Federal poverty line		
Above	3858 (87.2)	ref.
Below	440 (12.8)	**−0.74 (−0.92, −0.55)**
Census region		
South	1001 (34.0)	ref.
Midwest	1053 (22.7)	0.08 (−0.10,0.26)
Northeast	473 (18.6)	0.15 (−0.08,0.37)
West	1771 (24.8)	0.06 (−0.11,0.22)

Notes: All percentages are weighted. Bold font indicates statistical significance. Parameter estimates represent unstandardized coefficients.

### Trajectory of drinking days over time

Differences in the number of drinking days on selected dates, compared to March 11, 2020, are reported in Table 2. Compared to March 11, 2020, on average, US adults overall reported 0.36 (95% CI = 0.30, 0.43) more drinking days on April 1, 2020, 0.55 (95% CI = 0.47, 0.63) more drinking days on May 1, 2020, 0.41 (95% CI = 0.33, 0.49) more drinking days on June 1, 2020, and 0.39 (95% CI = 0.31, 0.48) more drinking days on July 1, 2020.

**Table 2:** Differences in the number of reported drinking days on different dates in the survey period, compared to March 11, 2020, overall and stratified by sociodemographic characteristics, among US adults in the UAS Panel, 2020 (*n* = 4298)

Population	Mean number of drinking days in the past week on March 11	Difference in number of drinking days^a^, β (95% CI)				*P* value for interaction^b^
		04/01	05/01	06/01	07/01	
Overall	1.82	**0.36 (0.30,0.43)**	**0.55 (0.47,0.63)**	**0.41 (0.33,0.49)**	**0.39 (0.31,0.48)**	N/A
Sex						
Male	2.16	**0.36 (0.27,0.45)**	**0.59 (0.48,0.71)**	**0.52 (0.41,0.63)**	**0.51 (0.39,0.63)**	**0.005**
Female	1.48	**0.37 (0.29,0.45)**	**0.50 (0.39,0.62)**	**0.29 (0.18,0.40)**	**0.27 (0.16,0.39)**	
Age (in years)						
18–29	0.86	**0.41 (0.20,0.61)**	**0.44 (0.19,0.69)**	0.10 (−0.17,0.37)	0.18 (−0.11,0.48)	**< 0.001**
30–49	1.72	**0.42 (0.31,0.52)**	**0.62 (0.47,0.76)**	**0.41 (0.28,0.54)**	**0.35 (0.21,0.49)**	
50–64	1.83	**0.37 (0.26,0.48)**	**0.56 (0.42,0.70)**	**0.43 (0.30,0.56)**	**0.44 (0.29,0.58)**	
65+	2.57	**0.22 (0.12,0.31)**	**0.44 (0.29,0.59)**	**0.53 (0.37,0.69)**	**0.54 (0.37,0.70)**	
Race						
White	1.99	**0.41 (0.35,0.48)**	**0.61 (0.52,0.70)**	**0.48 (0.39,0.57)**	**0.51 (0.42,0.61)**	**< 0.001**
Black	1.83	**0.28 (0.05,0.51)**	**0.44 (0.15,0.73)**	0.24 (−0.02,0.50)	0.06 (−0.24,0.37)	
Hispanic/Latino	1.40	**0.38 (0.20,0.57)**	**0.56 (0.29,0.82)**	0.26 (−0.01,0.53)	0.22 (−0.06,0.49)	
Other	1.22	0.03 (−0.22,0.28)	0.16 (−0.15,0.47)	0.25 (−0.04,0.53)	0.16 (−0.13,0.46)	
Household structure						
With partner only	2.31	**0.30 (0.21,0.40)**	**0.56 (0.43,0.70)**	**0.56 (0.43,0.68)**	**0.51 (0.38,0.65)**	**< 0.001**
Alone	1.84	**0.35 (0.20,0.51)**	**0.53 (0.34,0.72)**	**0.46 (0.28,0.63)**	**0.42 (0.23,0.62)**	
With partner and kids	1.55	**0.52 (0.40,0.63)**	**0.73 (0.56,0.89)**	**0.45 (0.30,0.60)**	**0.48 (0.33,0.64)**	
With kids only	2.02	0.17 (−0.12,0.46)	0.20 (−0.28,0.69)	0.07 (−0.43,0.56)	−0.07 (−0.58,0.44)	
Other	1.38	**0.32 (0.17,0.46)**	**0.40 (0.21,0.59)**	0.17 (−0.02,0.37)	0.20 (−0.01,0.40)	
Federal poverty line						
Above	1.82	**0.38 (0.32,0.44)**	**0.58 (0.49,0.67)**	**0.46 (0.38,0.54)**	**0.47 (0.38,0.55)**	**0.003**
Below	1.86	**0.25 (0.03,0.47)**	**0.32 (0.06,0.59)**	0.03 (−0.25,0.32)	−0.13 (−0.42,0.17)	
Census region						
South	1.80	**0.40 (0.29,0.51)**	**0.55 (0.41,0.69)**	**0.35 (0.21,0.49)**	**0.32 (0.16,0.48)**	**0.004**
Midwest	1.70	**0.26 (0.15,0.38)**	**0.47 (0.31,0.63)**	**0.39 (0.24,0.53)**	**0.43 (0.28,0.58)**	
Northeast	2.00	**0.30 (0.15,0.46)**	**0.47 (0.24,0.70)**	**0.43 (0.21,0.65)**	**0.42 (0.21,0.64)**	
West	1.83	**0.46 (0.35,0.56)**	**0.68 (0.54,0.82)**	**0.48 (0.34,0.62)**	**0.45 (0.29,0.61)**	

^a^ Reference is the number of drinking days on March 11, 2020.

^b^ Interaction terms are between the splines for days since March 10, 2020 and each sociodemographic characteristic. Bold font indicates statistical significance.

### Trajectories of drinking days over time among sociodemographic subgroups

**Figure 1:**
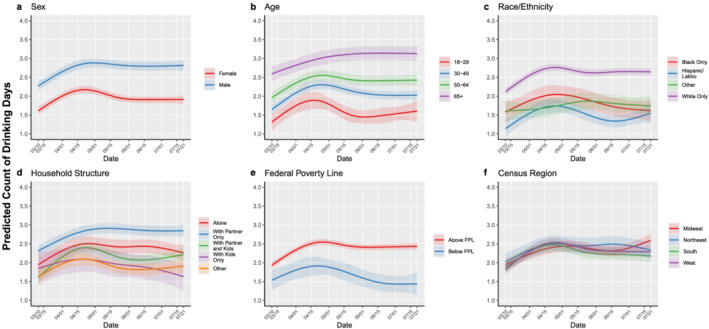
Predicted probabilities (bold lines) of self‐reported number of past week drinking days with 95% CI (shaded areas) by date of survey completion, stratified by different sociodemographic characteristics, among US adults in the UAS Panel, 2020 (*n* = 4298).

Results for each sociodemographic subgroup are displayed in Table 2. The predicted number of drinking days and 95% CIs on each day of the survey period, for each sociodemographic subgroup, are displayed in Fig. 1. Interactions between survey date and each covariate (sex, age, race/ethnicity, household structure, FPL, and census region) were statistically significant, indicating that trajectories of drinking days differed between sociodemographic subgroups. Both male and female participants reported more drinking days over time; however, in the latter half of the survey period, increases in drinking days attenuated among female (June 1: β = 0.29; 95% CI = 0.18, 0.40; July 1: β = 0.27; 95% CI = 0.16, 0.39), but not male participants (June 1: β = 0.52; 95% CI = 0.41, 0.63; July 1: β = 0.51; 95% CI = 0.39, 0.63). All age groups engaged in a greater number of drinking days in the first half of the survey period; by the latter half, adults ages 18 to 29 no longer engaged in a greater number of drinking days relative to baseline (June 1: β = 0.10; 95% CI = −0.17, 0.37; July 1: β = 0.18; 95% CI = −0.11, 0.48), whereas a sustained increase was observed among adults ages 65+ (June 1: β = 0.53; 95% CI = 0.37, 0.69; July 1: β = 0.54; 95% CI = 0.37, 0.70). For race/ethnicity, increases in drinking days were the largest in magnitude, and sustained over time, among White participants (April 1: β = 0.41; 95% CI = 0.35, 0.48; May 1: β = 0.61; 95% CI = 0.52, 0.70; June 1: β = 0.48; 95% CI = 0.39, 0.57; July 1: β = 0.51; 95% CI = 0.42, 0.61) compared to Black, Hispanic/Latino, and other racial/ethnic groups. For household structure, sustained increases in drinking days were observed among those living with a partner only, alone, or with a partner and children, whereas drinking days returned to a level comparable to baseline for those living with children only or in other household structures. A sustained increase in drinking days was observed for people living above the FPL, whereas drinking days for those living below the FPL returned to a level comparable to baseline in the latter half of the survey period (June 1: β = 0.03; 95% CI = −0.25, 0.32; July 1: β = −0.13; 95% CI = −0.42, 0.17). Increases in drinking days were observed across all regions, with slightly varying magnitudes over time.

### Sensitivity analysis

There were sociodemographic differences observed between those in the full sample compared to those included in the analytic sample with respect to sex, age, race, household structure, and poverty status (see Supporting information Table S3). However, the pattern of results in the complete sample of drinkers and non‐drinkers was broadly similar to the main analyses (see Supporting information Table S4), suggesting that our findings were not sensitive to the exclusion of non‐drinkers.

## Discussion

In this study, we examined longitudinal changes in number of drinking days in the past 7‐days among a nationally‐representative sample of US adults who reported any alcohol use between March 10, 2020 and July 21, 2020. We found that, in the overall sample, the number of drinking days appeared to peak in early May and remained significantly elevated through July 1, 2020, compared to March. Although some sociodemographic subgroups experienced decreases in the number of drinking days after an initial increase, other groups—including males; older adults; those living with a partner only, alone, or with a partner and children; those living above the FPL; and White respondents—had sustained increases in drinking days over time. This observed split response in trends of drinking behavior is consistent with evidence from other studies that have found that some sociodemographic subgroups have decreased alcohol consumption, whereas others have increased [14, 15, 27, 28, 29].

Although we observed significant increases in drinking days among the overall sample and multiple sociodemographic subgroups, these observed changes were small in absolute terms, corresponding to differences of less than one drinking day. However, this reflects significant percent increases (from baseline) ranging from 9%–51%. Furthermore, it is important to note that number of drinking days in isolation may yield an incomplete picture of changes in alcohol consumption, because we did not have consistent information on quantity of alcohol consumption (e.g. number of drinks per day), which could provide more context to these observed changes.

In previous research, certain sociodemographic characteristics have been associated with alcohol consumption [6, 7, 30, 31]. This is reflected in our findings, particularly with respect to increased alcohol consumption among males and older adults [6]. In the context of the COVID‐19 pandemic, one study showed increases in drinking behavior among males in April compared to February [19]. Our study expands on these findings, showing increases in the number of drinking days among both males and females, but that remained elevated over time for males and attenuated slightly for females. The attenuation in the number of drinking days among females could be a result of differences in coping abilities or strategies between these two groups [32].

Although all age groups demonstrated increases in the number of drinking days, this increase was sustained most notably among those ages 65 and older, a particularly vulnerable group related to adverse effects from social isolation [33]. Our finding contrasts those in other countries, where older adults were significantly less likely to report an increase in drinking [16, 17]. Older adults are at high risk for disability, morbidity, and mortality from alcohol‐related diseases, the prevalence of which have increased over the last decade [34]. Moreover, health risks related to alcohol use, such as suppression of immune functioning, could increase risk of COVID‐19 infection or complications from the virus, which is already at high risk of adverse health consequences because of COVID‐19.

Finally, there was a sustained increase in drinking days observed for those living above the FPL, whereas those living below the FPL returned to levels comparable to baseline. This finding is consistent with other studies who have observed increased alcohol consumption among those in higher income brackets [17]. Reductions in alcohol consumption among those with lower income may be because of decreased financial ability, particularly with the high rates of unemployment in the United States and delayed government response to provide consistent economic relief. Our findings within this subgroup analysis could also have implications for other observed trends, such as attenuation in drinking behavior among females and non‐White participants. The negative effects of the pandemic, including mortality, loss of employment/income, and psychological distress, have disproportionately affected racial/ethnic minorities and women [3, 35, 36], which could in turn limit access to alcohol because of stress and financial burden.

We recommend public health efforts, such as education, screening and surveillance, to support vulnerable subgroups and to avert both sustained alcohol consumption and potential transitions to problematic drinking. It is important to provide public health warnings about excessive alcohol consumption to prevent adverse effects of problematic alcohol use and to promote alternative positive coping strategies in response to stressful experiences. Although there are various motives for alcohol consumption, research has found that individuals who drink to cope in response to stress are at heightened risk for alcohol‐related problems [37, 38, 39]. Research from prior disasters and other stressful events has observed long‐term increases in drinking as a result of distress [40, 41, 42, 43]. There have been observed increases in mental distress and substance use to cope with the COVID‐19 pandemic [44]. It is imperative to consider the impact of COVID‐19 related stressors among the US population and monitor changes in risk behaviors, such as drinking, in response to these stressors.

It is also important to consider the environment in which individuals engage in alcohol consumption. With the closure of bars and restrictions on social gatherings, it is possible that there could be increased solitary drinking, which has been linked to symptoms of alcohol use disorder and other adverse mental health outcomes [45, 46, 47]. Our study found sustained increases in alcohol consumption among those who reported living alone and suggests that alcohol consumption within the context of COVID‐19 social distancing measures, particularly among those who may engage in solitary drinking, require further attention.

There are limitations of this study that are important to note. First, the survey did not collect data on the total number of standard drinks per drinking day. Therefore, we are unable to examine the prevalence of binge drinking and potential changes in the quantity of alcohol consumption. Second, survey dates were not randomly assigned at the first wave of data collection, and differences among participants who responded on earlier dates compared to those who responded on later dates could bias the observed results, though based on our sensitivity analysis, we do not have evidence to suggest that this caused significant bias in our analysis. Third, there were a number of sociodemographic characteristics that are known to be related to drinking behavior that were not examined such as sexual or gender identity, or time‐varying covariates like employment status. Future research should examine changes in and trajectories of drinking behavior in these groups. Fourth, there were some sociodemographic differences observed between participants who responded to all surveys compared to those who missed at least one survey. To the extent that participants who missed at least one survey collection period differed in their trajectory of drinking behavior, this may have biased our findings. Fifth, there were sociodemographic differences observed between those included in the analytic sample (i.e. those who reported drinking any alcohol during the study period) compared to those who reported no alcohol use, which may have affected the representativeness of our sample. Finally, the study used measures of drinking behavior on March 11, 2020 as the baseline for comparison and it is possible that some changes in drinking behavior in response to the pandemic had already occurred before that date.

Throughout the COVID‐19 pandemic, frequency of drinking among US adults has increased, peaking in early May, and remained at increased levels through mid‐July. Increased levels of drinking days were observed in some sociodemographic subgroups, particularly among men, White adults, those above the federal poverty line, and older adults. Supportive efforts and resources to prevent short‐ and long‐term problematic alcohol consumption during and after the COVID‐19 pandemic should be targeted at the population at large, as well as selectively at key subgroups that are identified to be at higher risk. As the pandemic continues, monitoring of alcohol consumption, as well as the incidence of problem drinking and alcohol use disorder, will be important priorities for public health surveillance and research.

## Declaration of interests

All authors have completed the ICMJE uniform disclosure form at www.icmje.org/coi_disclosure.pdf and declare: J.T., F.K., and E.A.S. report grants from National Science Foundation during the conduct of the study; J.T. also reports grants from Johns Hopkins Alliance for a Healthier World, Capital Group during the conduct of the study; no other relationships or activities that could have appear to have influenced the submitted work. The manuscript's guarantor affirms that this manuscript is an honest, accurate, and transparent account of the study being reported; that no important aspects of the study have been omitted; and that any discrepancies from the study as planned (and, if relevant, registered) have been explained.

## Author contributions

**Courtney Nordeck:** Conceptualization; writing ‐ original draft; writing ‐ review & editing. **Kira Riehm:** Conceptualization; formal analysis; writing ‐ original draft; writing ‐ review & editing. **Emily Smail:** Formal analysis. **Calliope Holingue:** Formal analysis. **Jeremy Kane**: Writing ‐ review & editing. **Renee Johnson:** Writing ‐ review & editing. **Cindy Veldhuis:** Writing ‐ review & editing. **Luther Kalb:** Writing ‐ review & editing. **Elizabeth Stuart:** Methodology; writing ‐ review & editing. **Frauke Kreuter:** Writing ‐ review & editing. **Johannes Thrul:** Conceptualization; methodology.

## Supporting information


**Table S1** Number of observations for each survey date.
**Table S2** Pearson's χ^2^ comparisons between participants completing all 9 waves (*n* = 2673) and those completing 8 waves or fewer (*n* = 1625).
**Table S3** Comparison of drinkers and non‐drinkers on sociodemographic characteristics (*n* = 6605).
**Table S4** Differences in the number of drinking days on different dates in the survey period, compared to 03/11/2020, overall and stratified by sociodemographic characteristics, among US adult drinkers and non‐drinkers in the UAS Panel, 2020 (*n* = 6605).
**Figure S1** Flow diagram of response rates, proportion of observations from ever drinkers, and proportion of complete observations at each wave.
