# Supplementary material for: Changes in drinking days among United States adults during the COVID‐19 pandemic
Source: Addiction. 2021 Jul 12;117(2):331–40. doi: 10.1111/add.15622 (PMC8441933; doi:10.1111/add.15622)
Supplement: Supplementary file 1 — Table S1 Number of observations for each survey date. Table S2 Pearson's χ2 comparisons between participants completing all 9 waves (n = 2673) and those completing 8 waves or fewer (n = 1625). Table S3 Comparison of drinkers and non‐drinkers on sociodemographic characteristics (n = 6605). Table S4 Differences in the number of drinking days on different dates in the survey period, compared to 03/11/2020, overall and stratified by sociodemographic characteristics, among US adult drinkers and non‐drinkers in the UAS Panel, 2020 (n = 6605). Figure S1 Flow diagram of response rates, proportion of observations from ever drinkers, and proportion of complete observations at each wave. [file ADD-117-331-s001.docx]

**Supplementary Table 1.** Number of observations for each survey date.

| **Date** | **Number of Observations** |  | **Date** | **Number of Observations** |  | **Date** | **Number of Observations** |
| --- | --- | --- | --- | --- | --- | --- | --- |
| 03/10 | 240 |  | 05/01 | 259 |  | 06/22 | 293 |
| 03/11 | 1,430 |  | 05/02 | 270 |  | 06/23 | 316 |
| 03/12 | 548 |  | 05/03 | 263 |  | 06/24 | 168 |
| 03/13 | 473 |  | 05/04 | 303 |  | 06/25 | 133 |
| 03/14 | 263 |  | 05/05 | 284 |  | 06/26 | 137 |
| 03/15 | 130 |  | 05/06 | 270 |  | 06/27 | 150 |
| 03/16 | 321 |  | 05/07 | 264 |  | 06/28 | 165 |
| 03/17 | 159 |  | 05/08 | 267 |  | 06/29 | 306 |
| 03/18 | 94 |  | 05/09 | 257 |  | 06/30 | 268 |
| 03/19 | 64 |  | 05/10 | 258 |  | 07/01 | 320 |
| 03/20 | 151 |  | 05/11 | 293 |  | 07/02 | 276 |
| 03/21 | 57 |  | 05/12 | 276 |  | 07/03 | 286 |
| 03/22 | 47 |  | 05/13 | 279 |  | 07/04 | 261 |
| 03/23 | 44 |  | 05/14 | 290 |  | 07/05 | 276 |
| 03/24 | 31 |  | 05/15 | 261 |  | 07/06 | 387 |
| 03/25 | 76 |  | 05/16 | 257 |  | 07/07 | 309 |
| 03/26 | 20 |  | 05/17 | 257 |  | 07/08 | 294 |
| 03/27 | 18 |  | 05/18 | 290 |  | 07/09 | 272 |
| 03/28 | 18 |  | 05/19 | 294 |  | 07/10 | 299 |
| 03/29 | 36 |  | 05/20 | 272 |  | 07/11 | 246 |
| 03/30 | 16 |  | 05/21 | 262 |  | 07/12 | 246 |
| 03/31 | 62 |  | 05/22 | 276 |  | 07/13 | 295 |
| 04/01 | 160 |  | 05/23 | 257 |  | 07/14 | 304 |
| 04/02 | 193 |  | 05/24 | 270 |  | 07/15 | 255 |
| 04/03 | 223 |  | 05/25 | 261 |  | 07/16 | 258 |
| 04/04 | 218 |  | 05/26 | 290 |  | 07/17 | 260 |
| 04/05 | 220 |  | 05/27 | 285 |  | 07/18 | 247 |
| 04/06 | 237 |  | 05/28 | 268 |  | 07/19 | 252 |
| 04/07 | 249 |  | 05/29 | 277 |  | 07/20 | 279 |
| 04/08 | 241 |  | 05/30 | 250 |  | 07/21 | 280 |
| 04/09 | 242 |  | 05/31 | 270 |  |  |  |
| 04/10 | 237 |  | 06/01 | 286 |  |  |  |
| 04/11 | 253 |  | 06/02 | 268 |  |  |  |
| 04/12 | 266 |  | 06/03 | 266 |  |  |  |
| 04/13 | 278 |  | 06/04 | 253 |  |  |  |
| 04/14 | 272 |  | 06/05 | 292 |  |  |  |
| 04/15 | 238 |  | 06/06 | 255 |  |  |  |
| 04/16 | 279 |  | 06/07 | 273 |  |  |  |
| 04/17 | 278 |  | 06/08 | 284 |  |  |  |
| 04/18 | 262 |  | 06/09 | 280 |  |  |  |
| 04/19 | 258 |  | 06/10 | 279 |  |  |  |
| 04/20 | 299 |  | 06/11 | 268 |  |  |  |
| 04/21 | 285 |  | 06/12 | 259 |  |  |  |
| 04/22 | 268 |  | 06/13 | 246 |  |  |  |
| 04/23 | 278 |  | 06/14 | 246 |  |  |  |
| 04/24 | 273 |  | 06/15 | 298 |  |  |  |
| 04/25 | 255 |  | 06/16 | 283 |  |  |  |
| 04/26 | 276 |  | 06/17 | 260 |  |  |  |
| 04/27 | 312 |  | 06/18 | 246 |  |  |  |
| 04/28 | 272 |  | 06/19 | 255 |  |  |  |
| 04/29 | 274 |  | 06/20 | 248 |  |  |  |
| 04/30 | 282 |  | 06/21 | 281 |  |  |  |

| **Supplementary Table 2.** Pearson’s chi-squared comparisons between participants completing all 9 waves (n=2,673) and those completing 8 waves or fewer (n=1,625). | | | |
| --- | --- | --- | --- |
| **Variable** | **Completed 8 Waves or Fewer (n=1,625); N (%)** | **Completed 9 Waves (n=2,673);**  **N (%)** | **P-value** |
| Sex |  |  |  |
| Male | 646 (44.6) | 1,243 (54.4) | **< 0.01** |
| Female | 979 (55.4) | 1,430 (45.6) |  |
| Age |  |  |  |
| 18-29 | 283 (19.4) | 239 (9.1) | **< 0.01** |
| 30-49 | 735 (49.4) | 917 (37.5) |  |
| 50-64 | 405 (21.0) | 856 (30.0) |  |
| 65+ | 202 (10.2) | 661 (23.5) |  |
| Race |  |  |  |
| White | 976 (57.5) | 1,934 (68.9) | **< 0.01** |
| Black | 124 (11.9) | 183 (10.9) |  |
| Hispanic/Latino | 349 (21.7) | 331 (12.8) |  |
| Other | 176 (9.0) | 225 (7.5) |  |
| Household Structure |  |  |  |
| With Partner Only | 415 (23.2) | 909 (34.0) | **< 0.01** |
| Alone | 236 (14.1) | 479 (17.1) |  |
| With Partner and Kids | 443 (29.3) | 634 (24.4) |  |
| With Kids Only | 74 (4.9) | 108 (3.9) |  |
| Other | 457 (28.4) | 543 (20.6) |  |
| Federal Poverty Line |  |  |  |
| Above | 1,438 (84.1) | 2,420 (89.2) | **< 0.01** |
| Below | 187 (16.0) | 253 (10.8) |  |
| Census Region |  |  |  |
| South | 370 (34.4) | 631 (33.7) | 0.46 |
| Midwest | 373 (23.2) | 680 (22.3) |  |
| Northeast | 150 (16.9) | 323 (19.6) |  |
| West | 732 (25.5) | 1,039 (24.3) |  |
| Notes: All percentages are weighted. Bold font indicates statistical significance. | | | |

| **Supplementary Table 3.** Comparison of drinkers and non-drinkers on sociodemographic characteristics (n=6,605). | | | |
| --- | --- | --- | --- |
| **Variable** | **N (%)** | | **P-value for Chi-Square Test** |
|  | **Drinkers  (n=4,298)** | **Non-Drinkers (n=2,307)** |  |
| Sex |  |  |  |
| Male | 1,889 (44.0) | 865 (37.5) | **< 0.001** |
| Female | 2,409 (56.0) | 1,442 (62.5) |  |
| Age |  |  |  |
| 18-29 | 522 (12.2) | 217 (9.4) | **< 0.001** |
| 30-49 | 1,652 (38.4) | 757 (32.8) |  |
| 50-64 | 1,261 (29.3) | 733 (31.8) |  |
| 65+ | 863 (20.1) | 600 (26.0) |  |
| Race |  |  |  |
| White | 2,910 (67.7) | 1,462 (63.4) | **< 0.001** |
| Black | 307 (7.1) | 193 (8.4) |  |
| Hispanic/Latino | 680 (15.8) | 369 (16.0) |  |
| Other | 401 (9.3) | 283 (12.3) |  |
| Household Structure |  |  |  |
| With Partner Only | 1,324 (30.8) | 422 (18.3) | **< 0.001** |
| Alone | 715 (16.6) | 655 (28.4) |  |
| With Partner and Kids | 1,077 (25.1) | 494 (21.4) |  |
| With Kids Only | 182 (4.2) | 101 (4.4) |  |
| Other | 1,000 (23.3) | 635 (27.5) |  |
| Federal Poverty Line |  |  |  |
| Above | 3,858 (89.8) | 1,900 (82.4) | **< 0.001** |
| Below | 440 (10.2) | 407 (17.6) |  |
| Census Region |  |  |  |
| South | 1,001 (23.3) | 690 (29.9) | **< 0.001** |
| Midwest | 1,053 (24.5) | 490 (21.2) |  |
| Northeast | 473 (11.0) | 232 (10.1) |  |
| West | 1,771 (41.2) | 895 (38.8) |  |
| Notes: Bold font indicates statistical significance. | | | |

| **Supplementary Table 4.** Differences in the number of drinking days on different dates in the survey period, compared to 03/11/2020, overall and stratified by sociodemographic characteristics, among US adult drinkers and non-drinkers in the UAS Panel, 2020 (n=6,605). | | | | | | |
| --- | --- | --- | --- | --- | --- | --- |
| **Population** | **Mean Number of Drinking Days in the Past Week on March 11** | **Difference in Frequency of Alcohol Consumption^a^, β (95% CI)** | | | | **P-value for Interaction^b^** |
|  |  | **04/01** | **05/01** | **06/01** | **07/01** |  |
| Overall | 1.14 | **0.24 (0.20,0.27)** | **0.36 (0.30,0.41)** | **0.26 (0.21,0.32)** | **0.26 (0.20,0.31)** | N/A |
| Sex |  |  |  |  |  |  |
| Male | 1.41 | **0.24 (0.18,0.30)** | **0.40 (0.32,0.48)** | **0.35 (0.27,0.43)** | **0.34 (0.26,0.42)** | **0.003** |
| Female | 0.89 | **0.23 (0.18,0.28)** | **0.31 (0.24,0.39)** | **0.18 (0.11,0.25)** | **0.17 (0.10,0.24)** |  |
| Age |  |  |  |  |  |  |
| 18-29 | 0.60 | **0.29 (0.15,0.43)** | **0.32 (0.15,0.49)** | 0.08 (-0.11,0.27) | 0.14 (-0.07,0.34) | **< 0.001** |
| 30-49 | 1.18 | **0.28 (0.21,0.35)** | **0.42 (0.32,0.52)** | **0.28 (0.19,0.37)** | **0.24 (0.15,0.33)** |  |
| 50-64 | 1.08 | **0.23 (0.16,0.30)** | **0.35 (0.26,0.45)** | **0.28 (0.19,0.36)** | **0.28 (0.18,0.37)** |  |
| 65+ | 1.41 | **0.13 (0.07,0.18)** | **0.26 (0.17,0.35)** | **0.31 (0.21,0.40)** | **0.31 (0.22,0.41)** |  |
| Race |  |  |  |  |  |  |
| White | 1.27 | **0.27 (0.23,0.31)** | **0.40 (0.34,0.46)** | **0.32 (0.26,0.37)** | **0.34 (0.28,0.40)** | **< 0.001** |
| Black | 1.13 | **0.18 (0.04,0.33)** | **0.29 (0.10,0.47)** | 0.16 (-0.01,0.33) | 0.05 (-0.14,0.24) |  |
| Hispanic/Latino | 0.91 | **0.25 (0.13,0.37)** | **0.37 (0.19,0.54)** | 0.18 (0.00,0.35) | 0.15 (-0.03,0.32) |  |
| Other | 0.65 | 0.02 (-0.12,0.16) | 0.10 (-0.08,0.27) | 0.14 (-0.01,0.30) | 0.10 (-0.07,0.26) |  |
| Household Structure |  |  |  |  |  |  |
| With Partner Only | 1.50 | **0.20 (0.14,0.27)** | **0.38 (0.29,0.47)** | **0.37 (0.29,0.46)** | **0.34 (0.25,0.43)** | **< 0.001** |
| Alone | 1.08 | **0.22 (0.12,0.31)** | **0.33 (0.21,0.45)** | **0.29 (0.18,0.40)** | **0.26 (0.14,0.38)** |  |
| With Partner and Kids | 1.07 | **0.35 (0.27,0.44)** | **0.50 (0.39,0.61)** | **0.31 (0.21,0.42)** | **0.33 (0.23,0.44)** |  |
| With Kids Only | 1.32 | 0.13 (-0.07,0.32) | 0.16 (-0.16,0.47) | 0.06 (-0.27,0.39) | -0.03 (-0.37,0.30) |  |
| Other | 0.78 | **0.19 (0.10,0.27)** | **0.24 (0.13,0.35)** | 0.11 (-0.01,0.22) | 0.12 (0.00,0.24) |  |
| Federal Poverty Line |  |  |  |  |  |  |
| Above | 1.16 | **0.25 (0.21,0.29)** | **0.39 (0.33,0.44)** | **0.30 (0.25,0.36)** | **0.31 (0.25,0.37)** | **< 0.001** |
| Below | 1.08 | **0.14 (0.02,0.26)** | **0.18 (0.04,0.33)** | 0.03 (-0.12,0.18) | -0.06 (-0.21,0.10) |  |
| Census Region |  |  |  |  |  |  |
| South | 1.01 | **0.23 (0.17,0.30)** | **0.32 (0.24,0.41)** | **0.20 (0.12,0.29)** | **0.19 (0.09,0.28)** | **0.003** |
| Midwest | 1.19 | **0.19 (0.11,0.27)** | **0.34 (0.23,0.45)** | **0.28 (0.18,0.38)** | **0.30 (0.20,0.41)** |  |
| Northeast | 1.33 | **0.20 (0.10,0.31)** | **0.31 (0.16,0.47)** | **0.29 (0.14,0.44)** | **0.28 (0.14,0.42)** |  |
| West | 1.18 | **0.31 (0.24,0.38)** | **0.46 (0.36,0.56)** | **0.33 (0.23,0.42)** | **0.30 (0.20,0.41)** |  |
| Notes. ^a^Reference is the frequency of alcohol consumption on 03/11/2020. ^b^Interaction terms are between the splines for days since 03/10/2020 and each sociodemographic characteristic. Bold font indicates statistical significance. | | | | | | |

**Supplementary Figure 1.** Flow diagram of response rates, proportion of observations from ever drinkers, and proportion of complete observations at each wave.

**
Note:** Complete observation indicates that data was available for all identified covariates at baseline (Wave 1 – March 10 through March 31).
